# Study on Control System of Integrated Unmanned Surface Vehicle and Underwater Vehicle

**DOI:** 10.3390/s20092633

**Published:** 2020-05-05

**Authors:** Hyunjoon Cho, Sang-Ki Jeong, Dae-Hyeong Ji, Ngoc-Huy Tran, Mai The Vu, Hyeung-Sik Choi

**Affiliations:** 1 Division of Mechanical Engineering, Korea Maritime and Ocean University, 727 Taejong-ro, Yeongdo-gu, Busan 49112, Korea; jooninkmou@kmou.ac.kr; 2 Maritime ICT R&D Center, Korea Institute of Ocean Science & Technology, 385 Haeyang-ro, Yeongdo-gu, Busan 49111, Korea; jeongsk313@kiost.ac.kr; 3 Marine Security and Safety Research Center, Korea Institute of Ocean Science & Technology, 385 Haeyang-ro, Yeongdo-gu, Busan 49111, Korea; jidae@kiost.ac.kr; 4 Dept of Control & Automation, Ho Chi Minh City University of Technology, 268 Ly Thuong Kiet St., Dist.10, Ho Chi Minh City, Vietnam; tnhuy@hcmut.edu.vn; 5 School of Intelligent Mechatronics Engineering, Sejong University, 209 Neungdong-ro, Gwangjin-gu, Seoul 05006, Korea; maithevu90@sejong.ac.kr

**Keywords:** unmanned surface vehicle (USV), unmanned underwater vehicle (UUV), integrated system, underwater cable, offshore field test

## Abstract

In this paper, in order to overcome certain limitations of previously commercialized platforms, a new integrated unmanned surface vehicle (USV) and unmanned underwater vehicle (UUV) platform connected via underwater cable capable of acquiring real-time underwater data and long-time operation are studied. A catamaran-type USV was designed to overcome the limitations of an ocean environment and to play the role as the hub of power supply and communication for the integrated platform. Meanwhile, the UUV was designed as torpedo-shaped to minimize hydrodynamic resistance and its hardware design was focused on processing and sending the underwater camera and sonar data. The underwater cable driven by a winch system was installed to supply power from the USV to the UUV and to transmit acquired data form underwater sonar sensor or camera. Different from other previously studied cooperation system of USVs and autonomous underwater vehicles (AUVs), the merit of the proposed system is real-time motion coordination control between the USV and UUV while transmitting large amount of data using the tether cable. The main focus of the study is coordination of the UUV with respect to the global positioning system (GPS) attached at USV and verification of its performance throughout field tests. Waypoint tracking control algorithm was designed and implemented on USV and relative heading, distance control for USV–UUV coordination was implemented to UUV. To ensure the integrity of the coordination control of the integrated platform, a study on accurate measurement system of the relative position between the USV and the UUV by using the GPS and the ultrashort baseline (USBL) device was performed. Individual tests were conducted to verify the performance of USBL and AHRS, which provide the position and heading data of UUV among the sensors mounted on the actual platform, and the effectiveness of the obtained sensor data is presented. Using the accurate measurement system, a number of field tests were conducted to verify the performance of the integrated platform.

## 1. Introduction

There has been a global increase in the scale of research on developing and operating ocean platforms for ocean surveys and underwater structure management, owing to geopolitical reasons such as importance of maritime transportation routes and safety of navigation, as well as energy-related reasons such as securing underwater resources [1]. In particular, in coastal areas where construction and human activities occur frequently, humans may find it difficult to dive beyond 30 m or work continuously for more than an hour. Hence, several countries around the world have been researching and developing unmanned ships and underwater robots to replace such manual tasks [2,3].

Typical water surface survey platforms include ship, unmanned surface vehicles (USVs) [4], and towfishes [5], whereas underwater survey platforms include remotely operated vehicles (ROVs), autonomous underwater vehicles (AUVs), and underwater gliders, as reported by Steven et al. [6]. A survey performed on the surface of water makes it possible to acquire the exact position of a platform by employing a global positioning system (GPS) [7]. Alternatively, underwater survey platforms have a common advantage in that they yield results with high resolution and reliability; hence, they can approach the exploration targets. However, in the case of underwater platforms, errors in the position and azimuth are likely to increase gradually even if inertial navigation is performed using expensive sensors; this is because GPS data cannot be accessed when using these platforms [8]. Furthermore, in the case of an AUV or a glider, there are constraints on real-time data communication and limitations in the operating time due to the limited energy of the battery [6]. In contrast, an ROV has the advantage of acquiring GPS data or supplying power, as it is operated on a manned ship; however, an ROV is expensive to operate and has limitations in terms of its movement in a broad area of the sea and when acquiring a wide range of underwater data.

Recently, platforms capable of combining underwater and water surface platforms have been evaluated to address these problems and to expand the survey area of underwater platforms. For example, studies on cooperating USV and AUV were performed. Sarda et al. proposed a platform that employs thin tether cables in a form that combines a commercial USV and AUV, to launch and recover (L&R) an AUV [9]. However, real-time communication or power transmission between the USV and AUV is limited. Zwolak et al. studied a platform comprising an USV and AUV, similar to the previously mentioned configuration; this platform could L&R and can also acquire data from a wide underwater area [10]. In these types of platform, USV and AUV are combined to explore a wide range of the water and the position of the USV and the relative position of the AUV can be determined using GPS and hydroacoustic device. However, these platforms were associated with limitations in terms of real-time power supply and large underwater data transmission.

Only a few years ago, to compensate for the weak points of individual AUV, ROV, and the USV–AUV cooperative platform, studies on the USV and ROV cooperating system were performed. Lachaud, Emilie, et al. proposed a concept of combining USV–ROV and a communication analysis between the USV and ROV was performed [11]. Furthermore, a study on a small-sized USV and ROV observation system wasstudied, where the system was constructed using small-sized ROV and catamaran USV. It showed that the combined USV and ROV observation system has the potential to vastly increase the speed and range of inspection of the ROV. However, it does not have a coordination system between the USV and ROV, such that it cannot get accurate underwater images or sonar data [12]. Additionally, a study was performed for a surface–underwater combined platform using underwater cables. However, it focused mainly on the robot’s mathematical modeling and the underwater cable drag analysis without an actual platform or field test results [13]. There are also a few commercial platforms combining underwater and water surface platforms capable of acquiring real-time data via an underwater cable, but only the platform structure and operating system were introduced. The performance of the cooperating USV and UUV has not yet been presented [14,15].

In this paper, a new integrated USV and UUV platform connected by the underwater cable capable of acquiring real-time underwater data and long-time operation is studied. The main focus of the study is coordination of the UUV position with respect to the GPS attached at USV. For coordination of the integrated platform, a study on accurate measurement system of the relative position between the USV and the UUV by using the GPS and the ultrashort baseline (USBL) device was performed. Using the accurate measurement system, a tracking control of the UUV to the USV was implemented in field test, which is a new trial in the surface–underwater platform. For implementation of the cooperative motion of the platform, the structure and control system were constructed and designed. Furthermore, an integrated control algorithm of the surface–underwater platform was developed, and the performance of the developed integrated platform was verified through field tests.

The underwater cables provide stable and continuous power to the underwater platform such that it can perform tasks for extended periods and, consequently, acquire a large amount of underwater data in real time. In the integrated platform, USBL is a type of hydroacoustic position reference (HPR) system that is suitable for small platforms because the distance of the baseline is short, and the size of the measurement system is smaller than that of other HPR systems [16]. HPR systems can be used to compensate for errors that accumulate when conventional inertial navigation is employed; several previous studies have employed USBL to correct the position of an underwater platform. For example, Caiti et al. investigated the position of an AUV using a mixed LBL/USBL positioning method [17], and Ji et al. developed an algorithm to compensate for the position error using a USBL on a track-based robot for underwater construction [18].

**Figure 1:**
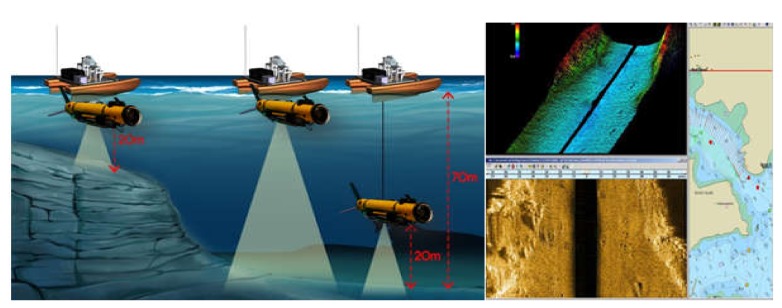
Operating concept and expected results of the integrated platform developed in this study.

The integrated platform developed in this study was aimed at achieving long hours of operation in the seacoast; acquiring a large amount of data and controlling the platform in real time; and accurately measuring the relative position between a USV and UUV using GPS and USBL, even after long hours of operation. Figure 1 presents the operating concept of the platform and expected results. By the winch and underwater cable, UUV can approach to inspection targets and receive sonar and video data in real time.

## 2. Configuration of Integrated Platform

### 2.1. Components of Integrated Platform

**Figure 2:**
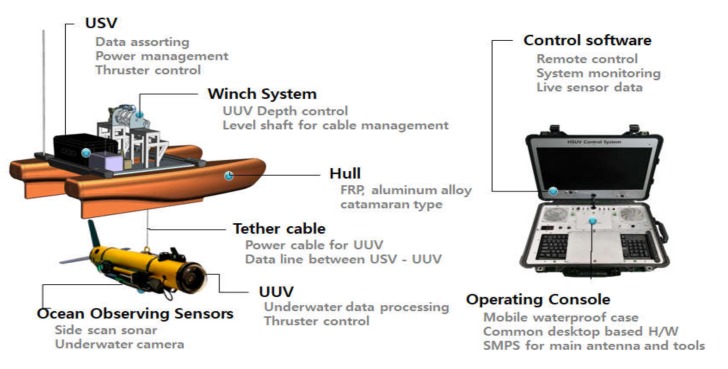
Components of the integrated system. USV, unmanned surface vehicle; UUV, unmanned underwater vehicle, operating console.

**Figure 3:**
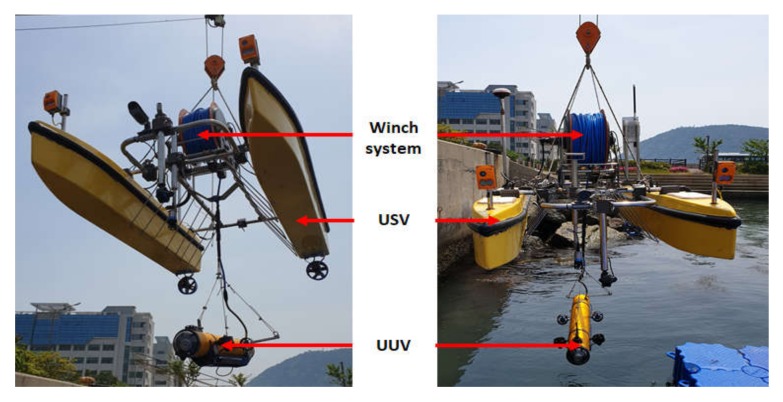
Configuration of the integrated platform.

The components of the integrated platform developed in this study are shown in Figure 2. The integrated platform mainly consists of a USV, a winch, an underwater cable, a UUV, and an operation console. The USV has a catamaran-type hull and comprises an integrated control system, three thrusters, and ocean observing sensors. The operation console for integrated management and control consists of a graphical user interface (GUI)-based operating system and commercial PC mainboard-based hardware for data acquisition and platform control. Figure 3 presents the exterior of the USV and the UUV of the fabricated integrated platform.

The USV performs three major roles in the integrated platform: (1) it serves as a communication hub for transmitting the sensor data of the USV as well as the sonar and image data acquired from the UUV of the integrated platform; (2) it manages the power supply of the entire system for operating the devices and actuators mounted on the platform and distributes power as required; and (3) it receives position information of the UUV in real time and provides control commands using the attached USBL, while performing waypoint tracking of the UUV.

The UUV performs two major roles in the integrated platform: (1) it processes data of the side scan sonar (SSS) and the underwater camera mounted for underwater exploration and sends the data to the USV; and (2) it detects the relative position and relative direction with respect to the USV using USBL to follow the USV and acquire underwater data based on GPS.

### 2.2. Structure of USV

**Figure 4:**
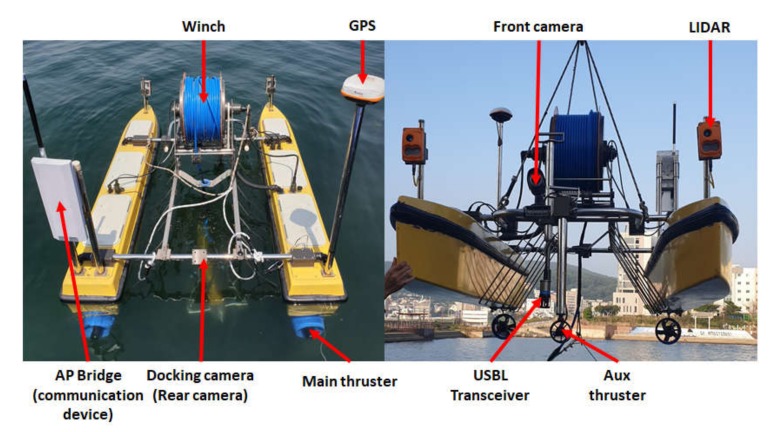
Assembled USV and component. GPS, global positioning system; USBL, ultrashort baseline.

The hull of the USV was fabricated as a catamaran-type composed of fiber-reinforced plastic material, considering the requirements of light weight and seawater-resistance. Communication devices, sensors, and a winch were installed on board for depth-control of the UUV, and batteries and control components were installed inside the hull. Figure 4 presents the exterior of the assembled USV; the total length and width of the hull are 2400 mm and 1400 mm, respectively.

### 2.3. Structure of UUV

**Figure 5:**
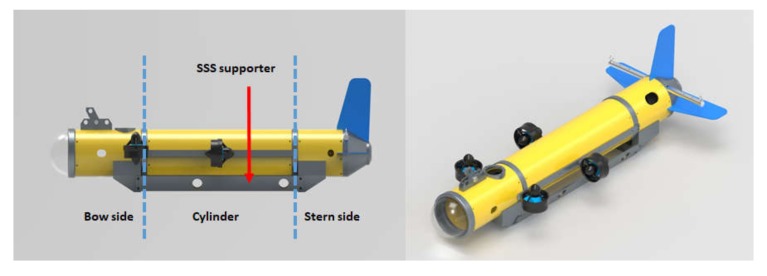
3D modeling of UUV. SSS, side scan sonar.

In this study, the UUV was designed to be torpedo-shaped to minimize water resistance. The hull is divided into three sections, namely, the bow side, pressure vessel, and stern side, as shown in Figure 5.

**Figure 6:**
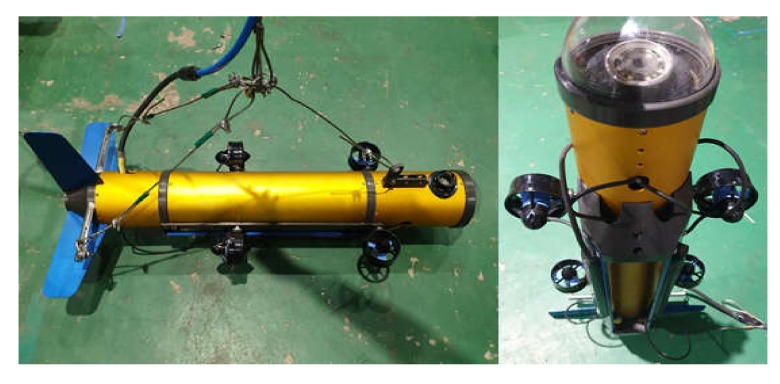
Photographs of assembled UUV.

The bow side consists of a transparent plastic nosecone for image capturing, underwater cable support, and USBL transponder. The pressure vessel has a single cylindrical structure containing control components. The stern side consists of a depth sensor, horizontal wings for attitude stability, tail wing, and underwater cable support. The exterior of the assembled UUV is shown in Figure 6.

**Figure 7:**
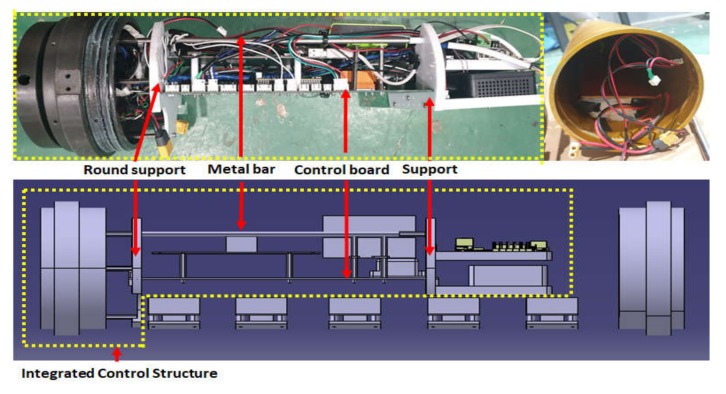
Configuration of inner structure of the cylinder.

Figure 7 shows the inner structure of the pressure vessel of the UUV. Five voltage conversion modules were fixed on the internal wall side at the lower part of the cylinder, in order to release heat generated by voltage conversion. All other components were designed to be mounted on the structure, which consists of a control board and round support (yellow dotted line in Figure 7) to facilitate easy mounting and dismounting.

### 2.4. Dynamics of Integrated Platform

**Figure 8:**
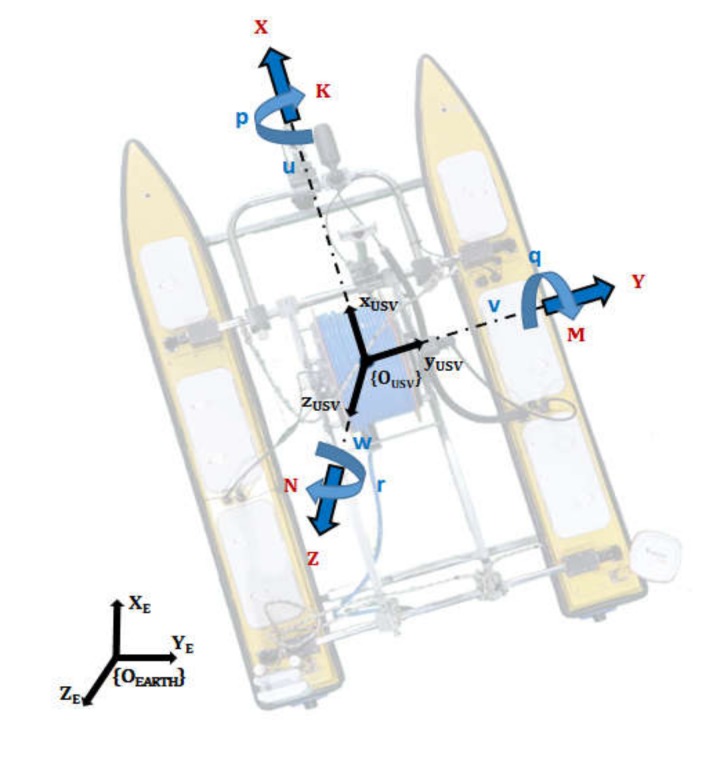
Earth-fixed and body-fixed coordinate system.

In the study, the integrated platform was composed of the USV, UUV, and connecting underwater cable. The vehicle dynamics of the integrated platform are presented. The frames of the earth-fixed and body-fixed are described as Figure 8, which is used to derive the dynamics of the USV on water surface.

The USV dynamics are expressed in accordance with the society of naval architects and marine engineers (SNAME) representation, which represents translational motion and rotational motion with actuating force and moments to the vehicle. The SNAME natation is expressed in Table 1.

The fundamentals of dynamics are based on the Newton–Euler formulation and vectorial mechanics. USV takes motion along the x and y axis, therefore, we can express the motion by surge, sway, and yaw. Furthermore, by considering the Coriolis-centripetal forces and damping, the dynamics of USV is derived and expressed as Equation (1). Detailed definition of the parameters and states can be referred in [19].

(1a) $$\left(m-X_{\dot{u}}\right)\dot{u}-\mu r+\left(Y_{\dot{v}}v_{r}r+Y_{\dot{r}}r-{\mathrm{mx}}_{G}r\right)r-\left(X_{u}+X_{u\left|u\right|}\left|u\right|\right)u\;=\;\tau_{X},$$

(1b) $$\left(m-Y_{\dot{v}}\right)\dot{v}+\left({\mathrm{mx}}_{G}-Y_{\dot{r}}\right)\dot{r}+\mu r-X_{\dot{u}}u_{r}r-\left(Y_{v}+Y_{v\left|v\right|}\left|v\right|\right)v-\left(Y_{r}+Y_{r\left|r\right|}\left|r\right|\right)r=\tau_{Y},$$

(1c) $$\left({\mathrm{mx}}_{G}-N_{\dot{v}}\right)\dot{v}+\left(I_{Z}-N_{\dot{r}}\right)\dot{r}-\left(Y_{\dot{v}}v_{r}r-Y_{\dot{r}}r+{\mathrm{mx}}_{G}r\right)u+X_{\dot{u}}u_{r}v-\left(N_{v}+N_{v\left|v\right|}\left|v\right|\right)v-\left(N_{r}+N_{r\left|r\right|}\left|r\right|\right)r=\tau_{N}.$$

Equation (1) represents the dynamics equation of the USV, where τ represents the propulsion forces from the two thrusters attached on USV.

In a similar approach, the UUV dynamics are expressed in Equation (2). In this study, since tracking of the UUV along the USV is the main issue, only the surge, sway, and yaw related with the relative position and relative heading angle are considered. The simplified UUV dynamics equations are described in Equation (2), where detailed equations including definition of the parameters and states can be referred in reference [19].

(2a) $$m_{f}[\dot{u_{f}}-v_{f}r_{f}+w_{f}q_{f}-x_{g_{f}}\left(q_{f}{}^{2}+r_{f}{}^{2}\right)+y_{g_{f}}\left(p_{f}q_{f}-\dot{r_{f}}\right)+z_{g_{f}}\left(p_{f}r_{f}+\dot{q_{f}}\right)=\sum X_{f},$$

(2b) $$m_{f}[\dot{v_{f}}-w_{f}p_{f}+u_{f}r_{f}-y_{g_{f}}\left(r_{f}{}^{2}+p_{f}{}^{2}\right)+z_{g_{f}}\left(q_{f}r_{f}-\dot{p_{f}}\right)+x_{g_{f}}\left(p_{f}q_{f}+\dot{r_{f}}\right)=\sum Y_{f},$$

(2c) $$I_{z_{f}}\dot{r}+\left(I_{y_{f}}-I_{x_{f}}\right)p_{f}q_{f}+m_{f}\left[x_{g_{f}}\left({\dot{v}}_{f}-w_{f}p_{f}+u_{f}r_{f}\right)-y_{g_{f}}\left(\dot{u}-v_{f}r_{f}+w_{f}p_{f}\right)\right]=\sum N_{f}.$$

The subscript f in Equation (2) is a symbol to avoid confusion with figures from USV. Sigma symbol on right side represents the sum of environmental forces and propulsion forces.

## 3. Integrated Platform Control System

### 3.1. USV

**Figure 9:**
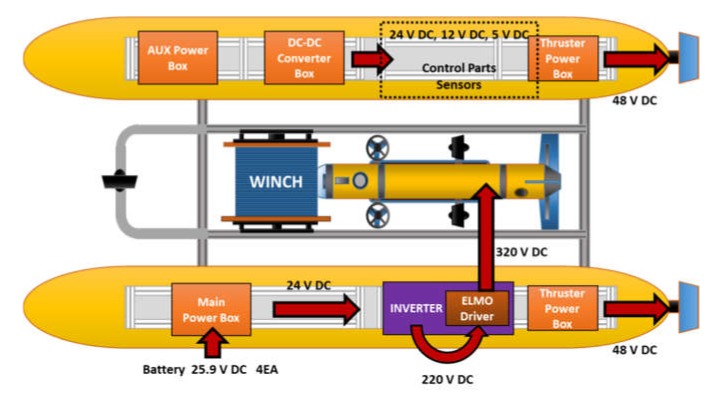
Diagram of USV power system.

**Figure 10:**
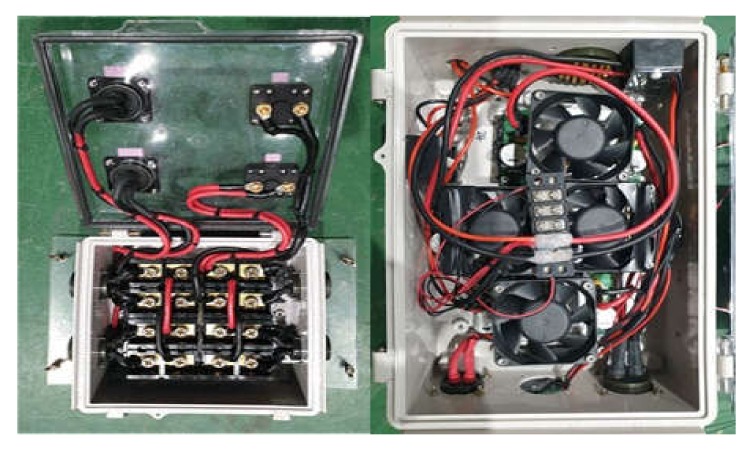
Power system control modules of USV.

Figure 9 shows a schematic diagram of the USV power system. The key power components are installed in a waterproof section for modularization and to protect against water leakage; all other components outside the module were equipped with built-in waterproof and emergency power shutoff functions. Figure 10 shows the fabricated power system control module.

**Figure 11:**
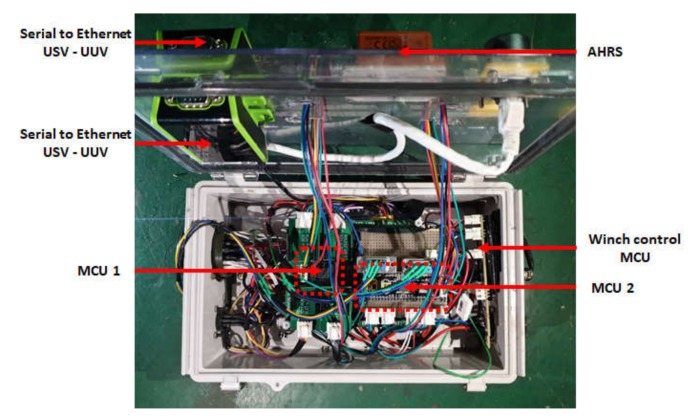
Control module of USV. AHRS, attitude heading reference system; MCU, micro processing unit.

**Figure 12:**
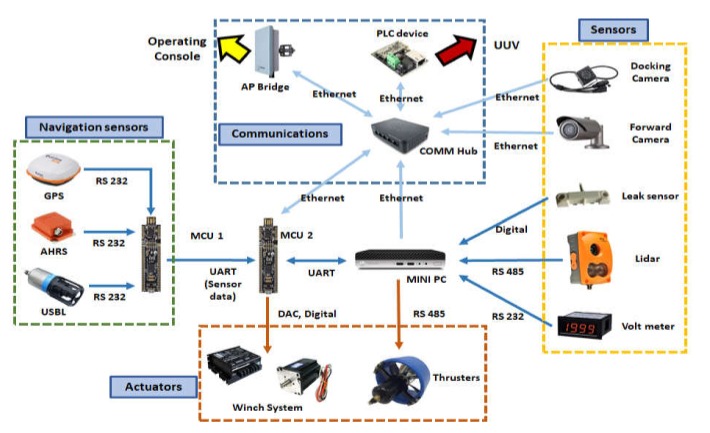
Diagram of USV control system.

A mini PC and a control module were mounted on the right side in the hull of the USV to enable control functions. The control module is a core component of the USV control system; it organizes sensor data, communicates with land, controls the USV, and sends UUV control commands. Figure 11 shows the internal configuration of the control module, and Figure 12 shows the relationships between the micro processing units (MCUs), mini PC, and peripheral devices.

Micro processing unit 1 (MCU1 or M1) receives and processes data from navigation sensors and sends the output to micro processing unit 2 (MCU2 or M2). M2 receives control commands transmitted from land via communication equipment and executes these control commands using sensor data received from M1.

### 3.2. UUV

**Figure 13:**
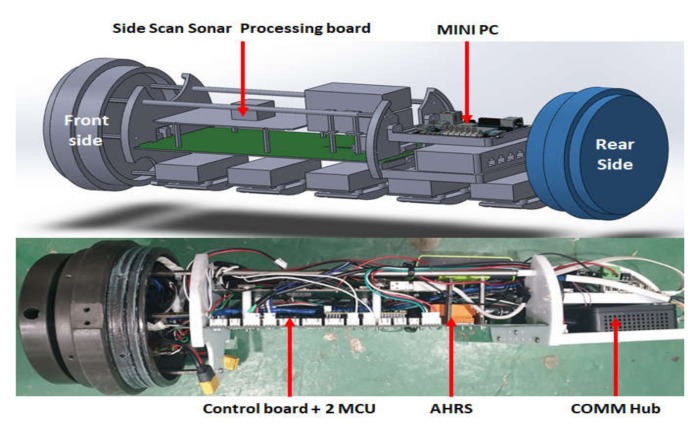
Configuration of UUV control system.

An SSS processing board, attitude heading reference system (AHRS), mini PC, and communication hub were mounted around the control board attached with two MCUs, which is the main processor, in the UUV. Figure 13 shows the configuration of the control components.

**Figure 14:**
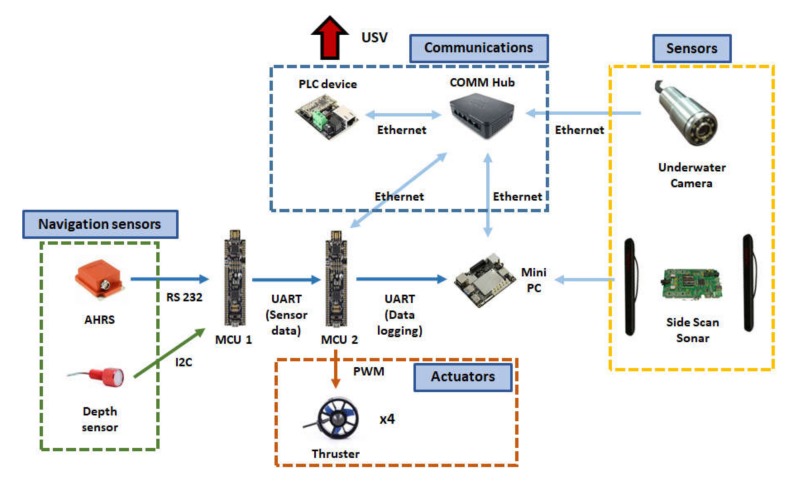
Diagram of UUV control system.

The control system of UUV consists mainly of two MCUs and one mini PC. MCU1 receives and processes data from navigation sensors and sends the output to MCU2. M2 uses sensor data received from M1 and navigation data received from the USV to execute control commands. Figure 14 shows the relationships between the MCUs, mini PC, and peripheral devices.

### 3.3. Operation Program

**Figure 15:**
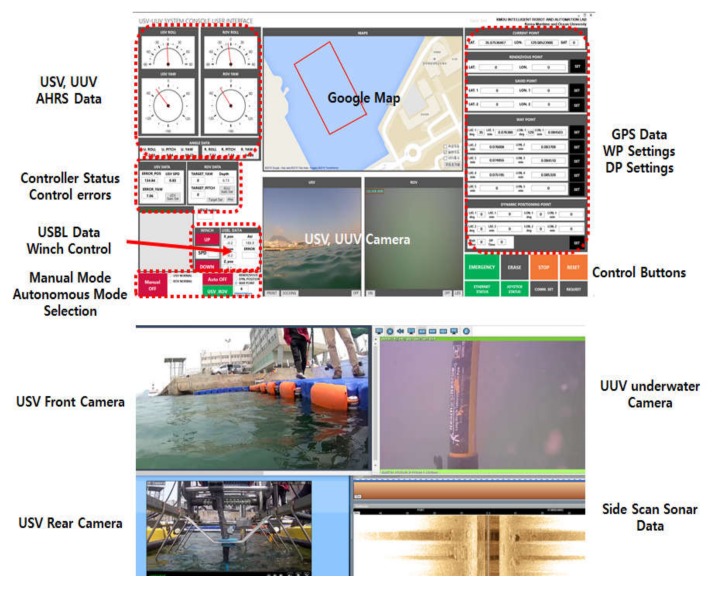
Operation program for the integrated system.

The operation program of the integrated platform was developed using the C# programming tool. Figure 15 shows the operation program. In the figure, the screen at the top shows the interface of the operation program, whereas the screen at the bottom shows real-time images and the SSS data display screen. The operation program displays numerical values such as the navigation sensors’ data, control gains, and control errors. Furthermore, it verifies the current position of the platform by interlinking with Google Map and can draw paths on the map following the input waypoints.

## 4. Combined Control System of Integrated Platform

**Figure 16:**
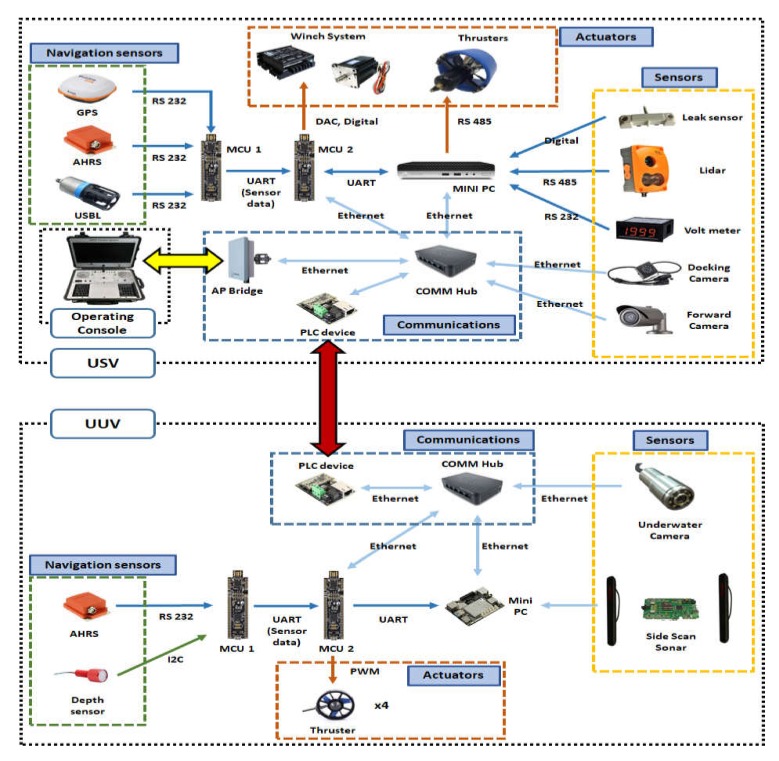
Diagram of the integrated platform control system.

Figure 16 shows the overall configuration diagram of the integrated platform control system. The integrated platform needs to process a large amount of data such as sonar data and image data between the operation console, USV, and UUV, and transmission control protocol/internet protocol (TCP/IP) communication was adopted to accomplish this. The control commands of the platform are sent and received in packets; in the case of USV-only control, the control command packet sent from the operation console is input to the MCU via the communication hub of the USV to control the USV. However, if the packet input to the USV is USV–UUV co-operative commands, USV will generate packets which include USV’s sensor data. Subsequently, it sends the control command to the UUV via the communication hub.

The integrated platform has to process a large amount of data such as sonar data and image data between the operation console, USV, and UUV, and TCP/IP communication is adopted to accomplish this. The control commands of the platform are sent and received in packets, and in the case of single command of USV, the control command packet sent from the operation console is input to the MCU of USV via the communication hub of USV to execute the control of USV. However, if the packet input to the USV is a cooperation command of USV and UUV, the USV generates packets for the USV and the UUV including the control command and the USV’s sensor data. Then, it sends the control command to the UUV via the communication hub.

In this study, the integrated platform’s control method is based on proportional integral differential (PID) controllers with modifications. There are advanced path tracking algorithms currently presented [20,21], but the goal of this combined controller is based on verifying the controllability based on experimental results. By adopting PID-based controller, it was possible to adjust the variables easily while performing field tests. Advanced path tracking control algorithm is considered for future research.

In this study, a leader–follower strategy was used for tracking UUV along the USV motion. When considering tracking of the UUV along the USV, sway motion can be neglected. In this manner, the control mission is to achieve the position error $\int e\left(u-u_{f}\right)\mathrm{dt}=0$ and the yaw angle error $\int e\left(r-r_{f}\right)\mathrm{dt}=0$.

**Figure 17:**
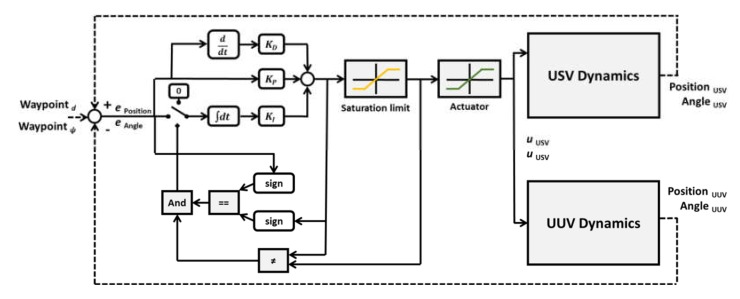
Diagram of the integrated platform coordination control flow.

For this, PID-based anti-windup control was applied. Figure 17 represents the coordination control between USV and UUV. In case of manual control operation, the USV moves first and the UUV follows to compensate the compared position error and the heading angle error, which is derived by the USV and the UUV dynamics.

### 4.1. USV’s Waypoint Tracking Control Algorithm

The USV’s path control is a control process in which the USV follows a path connecting the target waypoint coordinates. Path control consists of recursion of heading control and tracking control. Heading control is a method of controlling the hull direction in a position to match the desired heading angle obtained using the line of sight (LOS) method [22] and the direction of the USV’s bow. Tracking control is a process that enables tracking of the path connecting the target position and the current position after setting the heading.

This study considered straight driving and turning simultaneously when tracking control is performed to track the path between the waypoints. The control error for straight driving is input as distance, and the error for turning is input as heading angle. The two controllers for the PID-based distance and direction can be expressed as:

(3) $$T_{\psi }=K_{P_{\psi }}e_{\psi }+K_{I\psi }\int e_{\psi }+K_{D\psi }\dot{e_{\psi }},\;T_{d}=K_{P_{d}}e_{d}+K_{Id}\int e_{d}+K_{Dd}\dot{e_{d}}.$$

In Equation (3), T represents the output of the PID controller. As mentioned, while performing tracking control, both the heading angle and distance are considered as control error in order to enhance tracking performance. The output of the two main thrusters at the stern is controlled using the weight scheduling method, which assigns different weights to the powers of the two controllers. The weights were assigned to effectively allocate the power of the two controllers within the limited range of thruster control inputs. Different weights can be assigned at the starting stage of a movement that requires a larger input energy for acceleration or when smooth turning is performed. The weight scheduling method is described as:

(4) $$Input_{port}=sgn\left(R_{\theta }\right)\left[T_{\psi }W\right]+sgn\left(R_{\theta }\right)\left[T_{d}\left(1-W\right)\right],\;Input_{stbd}=sgn\left(R_{\theta }\right)\left[T_{\psi }W\right]+sgn\left(R_{\theta }\right)\left[T_{d}\left(1-W\right)\right]$$

**Figure 18:**
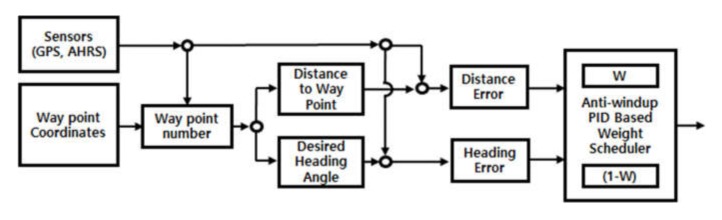
Diagram for tracking control.

In the above equation, $T_{\psi }$ and $T_{d}$ is the output of two different controllers, as shown in Equation (4) and $sgn\left(R_{\theta }\right)$ is the value that determines the sign of the relative angle between the desired heading angle and the platform. W represent the weight, the values which are between 0 to 1. Figure 18 shows a block diagram of the tracking control based on an anti-windup PID controller and Equation (4).

This control method includes an anti-windup scheme in the PID controller. Various schemes have been implemented for anti-windup, including the back-calculation method, anti-windup method, and restricted integral method [23,24]. This study used the anti-windup method employing the clamping method, which is easy to implement.

### 4.2. UUV’s Waypoint Tracking Control Algorithm

Heading angle tracking and the relative distance control method were employed in the integrated platform for UUV’s USV tracking control. Heading angle tracking is a method used for matching the heading angle of the UUV’s hull with the heading angle of the USV’s hull. It consists of a controller for matching the heading angle of the UUV based on the heading angle data of the USV and a controller for maintaining the posture of the UUV to achieve stable sensor data. The heading angle is controlled using two thrusters at the stern, and the attitude angle is controlled using two thrusters at the center of the hull.

**Figure 19:**
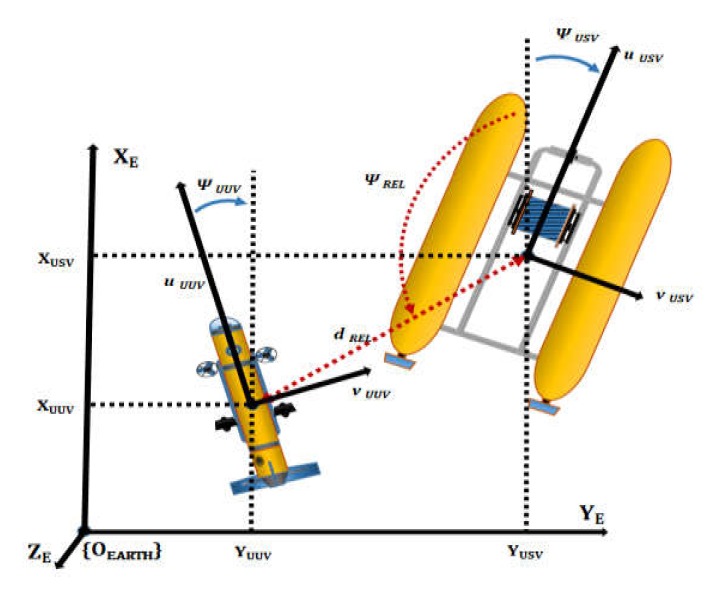
Graphical description of $d_{REL}$ and $\psi_{REL}$.

The relative distance control method uses the relative distance data $d_{REL}$ obtained from USBL relative position coordinate data and the relative heading $\psi_{REL}$ obtained from USBL azimuth data. The visual description of $d_{REL}$ and $\psi_{REL}$ is shown in Figure 19. Path control is performed, similar to the tracking control method of the USV, to rotate the hull direction of the UUV toward the origin point of the USBL (the coordinate of the USBL transceiver installed in the USV) and reduce the relative position toward the origin point of the USV. Here, the relative angle and relative position are continuously updated as the moving USV’s position and direction change continuously.

## 5. Performance Test of Integrated Platform

### 5.1. Sensor Performance Test

Position and heading angle information can be easily obtained for navigation on water; however, the position and heading angle information of the UUV is obtained using the USBL and AHRS in the integrated platform. The performance and reliability of the sensors need to be verified for accurate control of the integrated system.

#### 5.1.1. USBL Performance Test

**Figure 20:**
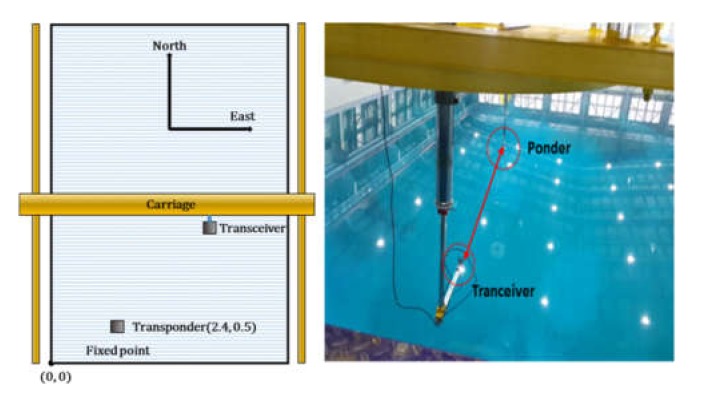
USBL test in engineering basin.

A USBL performance test was conducted in an ocean engineering basin. The USBL transponder (SeaTrac X010, Blueprintsubsea, Ulverston, UK) was anchored using a crane, and the transceiver (SeaTrac X150, Blueprintsubsea, Ulverston, UK) was attached to the carriage and moved in the X and Y directions to obtain the data. Figure 20 shows the equipment in the basin and the basin’s coordinate system used for the test.

**Figure 21:**
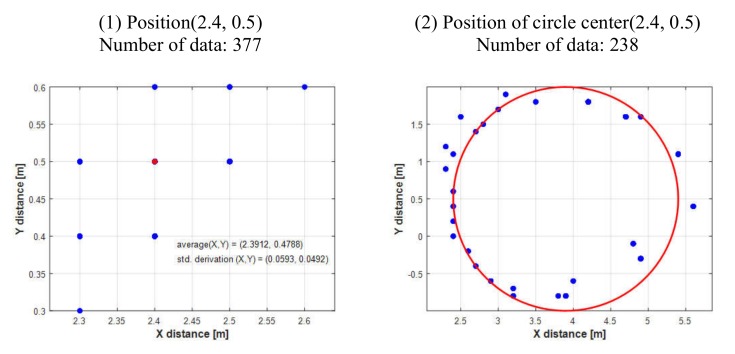
USBL test results.

The *y*-axis of the basin was set to the true north. The lower left of the basin was set to (0, 0), and the transponder was installed at (2.4 m, 0.5 m) coordinates. The location of the transceiver was moved, while maintaining a relative depth of 0.8 m between the USBL transponder and transceiver. Then, data were obtained in the stationary state and during circular movements. Figure 21 shows some of the acquired data. In the stationary test results, the errors in each axis were 0.88 cm and 2.12 cm, respectively. Many test values were obtained to confirm that reliable data can be obtained if the distance between the two devices does not deviate from the reaching range of the sonar radiation angle.

#### 5.1.2. AHRS Performance Test

The AHRS (MTI-30, Xsens, Enschede, The Netherlands) was attached to the integrated platform in order to obtain the attitude and heading data of the USV and UUV. In the test, the GPS (Hemisphere H200, Hemisphere GNSS, Scottsdale, AZ, USA) and AHRS were fixed on a straight-line structure and attached to face the same direction. Then, the process of comparing the sensor data obtained after setting various internal filters of AHRS was repeated to find the optimal values and verify the heading error.

**Figure 22:**
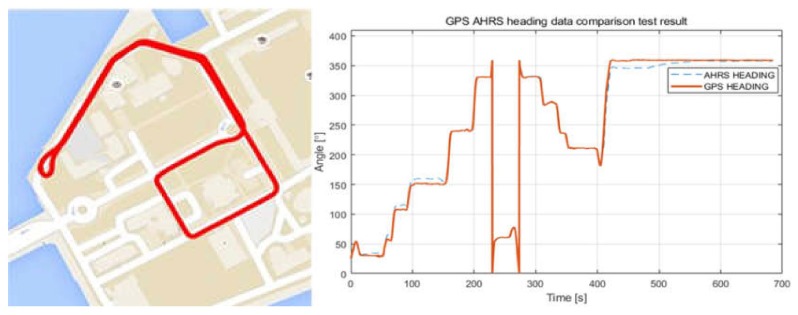
Result of GPS and AHRS test.

The test was conducted in the campus of Korea Maritime and Ocean University. The structure where the GPS and AHRS were fixed was attached to a vehicle and driven inside the campus to obtain GPS and AHRS data; the data were obtained using the program provided by the manufacturers without additional post-processing. The test results are shown in Figure 22.

The left side of Figure 22 shows the actual path followed by the GPS and AHRS attached to the vehicle, whereas the right side shows the heading angle acquired by the GPS and AHRS. Errors of less than 10° were observed for 100–150 s after driving commenced and after driving was completed. This range corresponds to the time delay caused by the algorithm of the geomagnetic compensation filter; an error of less than 1° was observed in other sections.

### 5.2. Offshore Field Test of Integrated Platform

The tests were conducted mainly in two locations, namely in the sea near the marina of Korea Maritime and Ocean University and near the external breakwater of Busan Port.

#### 5.2.1. Waypoint Tracking Control Test

**Figure 23:**
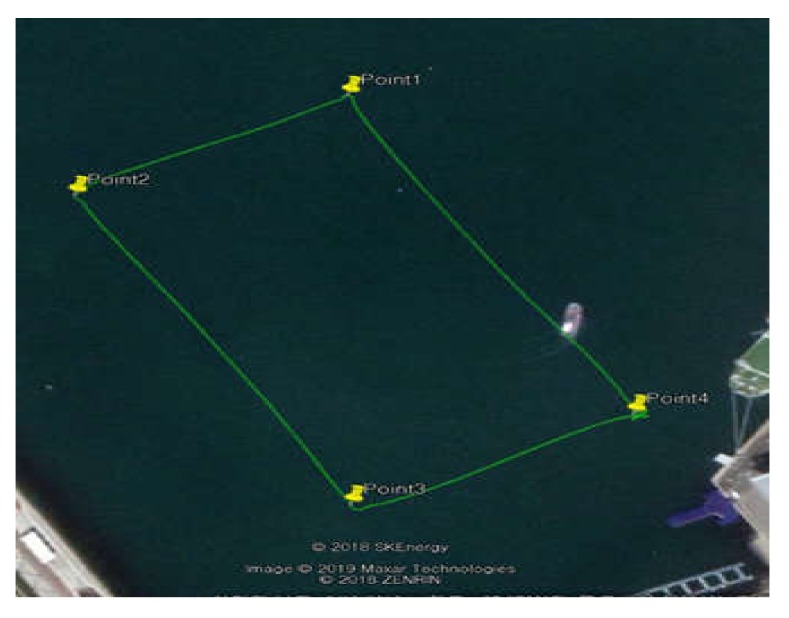
Waypoint tracking control results.

For waypoint tracking (WP), rectangular paths comprising four coordinates were tracked. The latitude and longitude details of these coordinates are listed in Table 2. Figure 23 shows the results of converting the latitude and longitude data into the universal transverse mercator (UTM) grid coordinate system using Google Map program.

**Figure 24:**
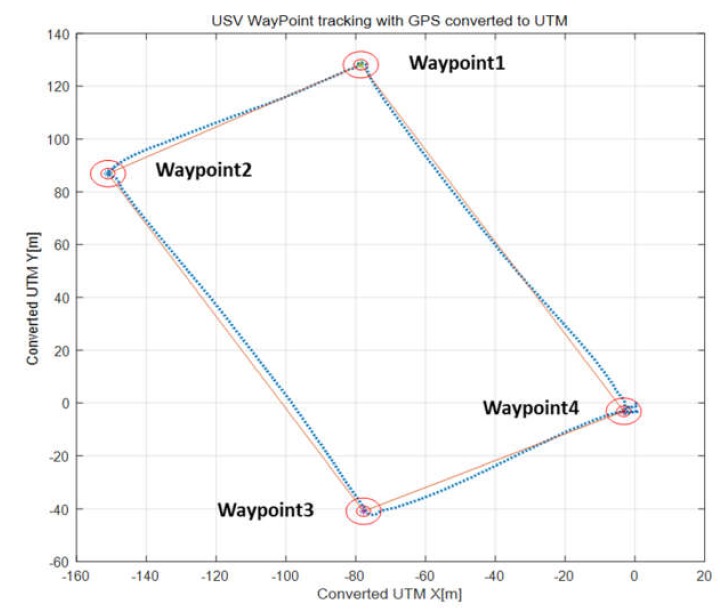
Trajectory during waypoint tracking. UTM, universal transverse mercator.

Waypoint tracking control was performed after submerging the UUV to a depth of 3 m as the depth of the seabed at the test location was less than 6 m at the lowest point. During waypoint tracking, the UUV tracked the heading angle of the USV and the maximum speed of the USV was limited to 2.5 kn in the test. According to data obtained from the Korea Hydrographic and Oceanographic Agency (KHOA), the tidal current of this area was estimated as 0.6–0.8 kn in the southwest direction, and the wind direction was south–southeast, whereas the wind speed was 4.0 kn on average and 6.5 kn at maximum [25]. Figure 24 shows the result of converting the position data into the UTM coordinate system; the figure shows the straight-line path between the waypoints and the moving path of the platform. The solid line represents the line of the desired path connecting the straight lines between the waypoints, whereas the dotted line represents the actual moving path of the platform. At waypoints 1 and 2, it was confirmed that the actual path of the USV turned inside a circle with a radius of 2 m. In the case of waypoint 3, turning occurred inside a radius of 5 m.

**Figure 25:**
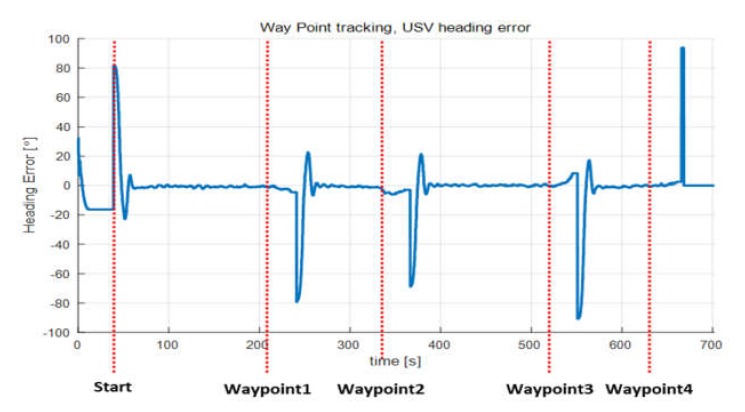
USV heading error during waypoint tracking.

Figure 25 shows the heading errors of the USV during waypoint tracking control. Heading error occurred only in the section where turning motion was performed. Turning was performed after a certain delay instead of turning immediately after reaching the waypoint because a delay time was set to depart to the next waypoint after the UUV had sufficiently advanced. The heading error while performing waypoint tracking control was calculated to be in the range of ±80–90°, as shown in Figure 25. The errors are close to a right angle because the input path was in a rectangular shape.

**Figure 26:**
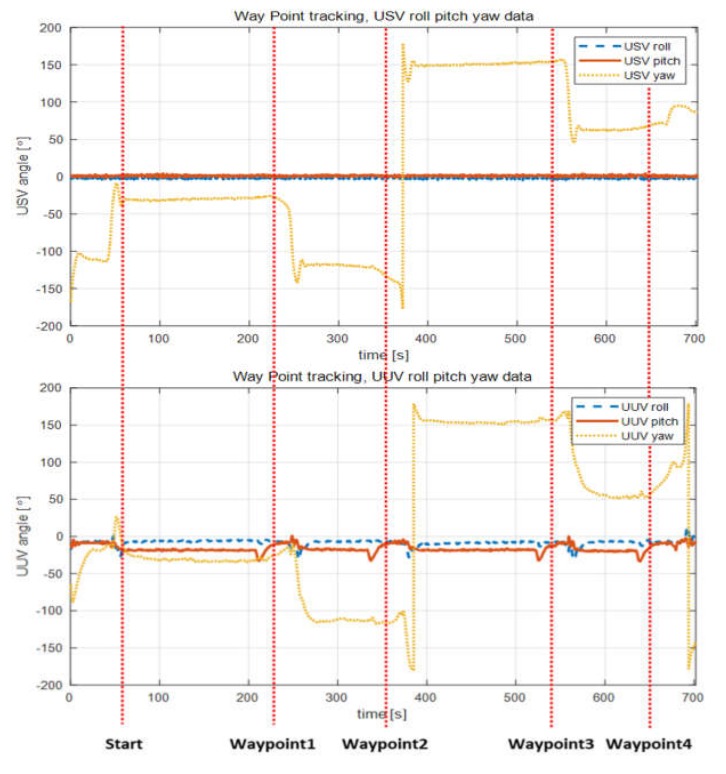
Attitude values of USV and UUV during waypoint tracking.

Figure 26 shows the attitude sensor data of the USV and UUV during waypoint tracking. Here, the dotted line represents the time of reaching each waypoint.

In the case of the USV, the roll and pitch angles during driving were stable from +3° to −3° without large fluctuations. The UUV maintained a certain roll and pitch, while the USV performed straight-line movement, verifying that attitude control was performed appropriately. The roll and pitch angles approached 0 while reaching and turning at the waypoints. This indicates that the attitude is nearly stable at 0° when the USV is turning at a fixed position because the tension produced by the underwater cable (i.e., the external force acting on the UUV) decreases.

**Figure 27:**
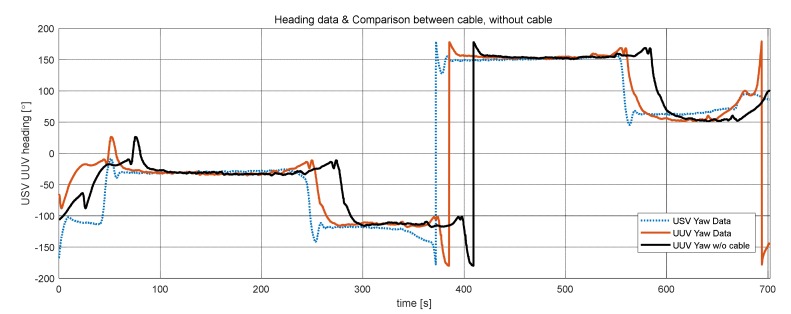
Heading data of USV and UUV during waypoint tracking and simulated result of the non-cabled collaborating system.

Figure 27 shows heading angles of the USV and UUV measured by the sensors while performing the waypoint tracking control shown in Figure 26. Analysis of the sensor heading angle data of the USV and UUV shown in Figure 27 confirmed that the UUV’s relative heading angle control was performed according to design specifications, while the USV performed waypoint tracking control.

The present USV–UUV collaboration system without underwater cable should transmit the camera and sonar images and navigation data between the USV and UUV using the acoustic communication device. However, the acoustic communication device is not capable of real-time communication due to slow transmission capability. For example, the data transmission capability of one of the best present acoustic communication devices (Modem 6 Standard, Sonardyne, Yateley, UK) is 9000 bps, in other words, 1.125 KB/s. The installed underwater camera (Sensor-6882, CharminTech, Bucheon, Republic of Korea) on the integrated platform requires 687 KB/s speed for transmitting the motion image 1920 × 1080 for 60 frames per seconds. Also, the side scan sonar (StarFish 453OEM, Blueprintsubsea, Ulverston, UK) requires approximately 24.24 KB/s transmission speed. For transmitting the data of the devices except navigational data, the side scan sonar requires about 22 s, while the camera requires 700 s, which means that it is impossible for the acoustic device to transmit the data of the underwater camera and sonar. However, the data transmission speed of the data using the underwater cable of the integrated platform is 11.04 MB/s, which is 9813 times faster than the expensive and bulky acoustic device and is enough for large data transmission.

For the case of using the side scan sonar in UUV tracking along the USV without underwater cable, the simulation result was performed and is presented in Figure 27 (black bold line). As shown in Figure 27, it seems very difficult for the UUV to track the USV since about 25 s of time delay including processing time occurs.

**Figure 28:**
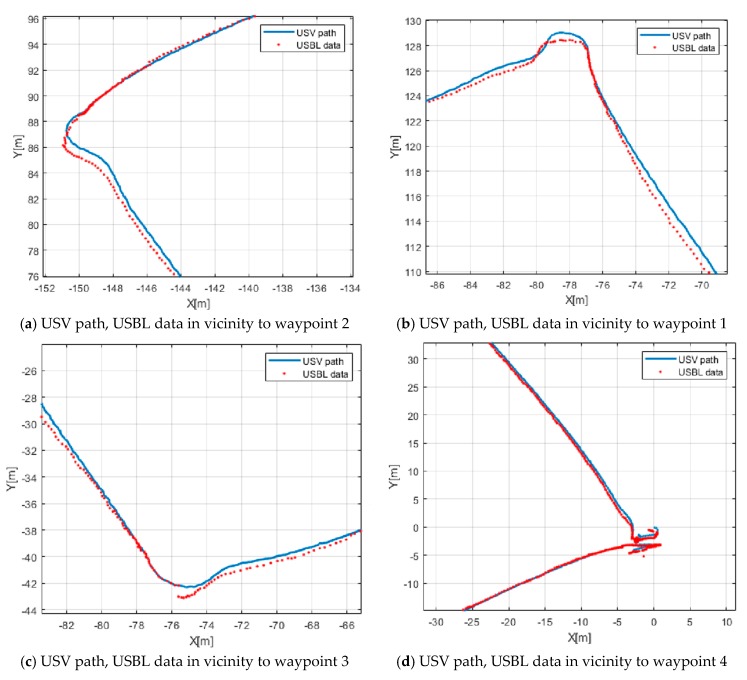
USV path and USBL data.

The degree of waypoint tracking between USV and UUV can be estimated using data received from the USBL. The X and Y data of the USBL were visualized simultaneously with the USV’s path converted into the UTM coordinate system, as shown in Figure 28.

Figure 28 shows the trajectory of the USV and UUV while tracking the four waypoints. It depicts the UUV’s path measured using the USBL, as well as the USV’s path around the four waypoints listed in Table 2. The difference between the paths of the USV and UUV was less than 2 m immediately after turning, but the paths of the USV and UUV coincided shortly afterward.

#### 5.2.2. Relative Distance Control Test

The UUV control methods are mainly divided into heading tracking control and relative distance control. A separate test was conducted to investigate the relative distance control performance. The depth of the test area was 20 m and the test was conducted by submerging the UUV to a depth of 17 m. The tidal current of this area was estimated as 0.7–0.93 kn in the southwest direction based on KHOA’s data, and the wind direction was south–south-southeast with average and maximum wind speeds of 41.5 kn and 6.5 kn, respectively [25].

**Figure 29:**
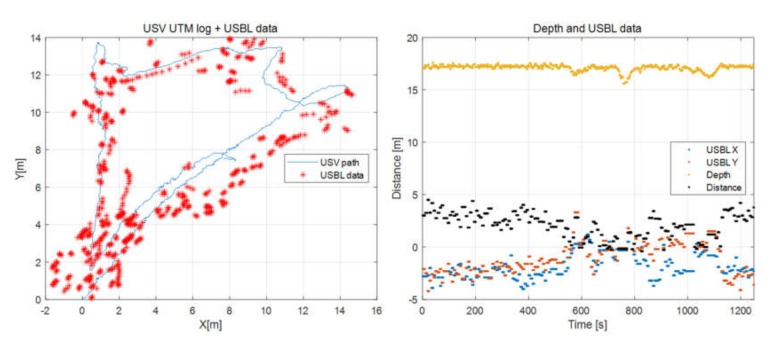
Relative distance control test result.

The test results are shown in Figure 29. The left side of the figure shows the USV’s moving path and the USBL coordinates. The right side shows data measured by the UUV’s depth sensor, X and Y data of the USBL, and the relative distance of the USV and UUV obtained using the received X and Y data (distance on the right side of Figure 29). Despite submerging the UUV to a depth of 17 m and moving the USV on an arbitrary path at 2.5–3 kn, the relative distance of the USV and UUV was controlled with errors of less than 5 m.

## 6. Conclusions

In this paper, in order to overcome typical disadvantages of previously commercialized ocean platforms, a new integrated maritime platform composed of a highly operable unmanned ship and an underwater vehicle capable of acquiring underwater data connected by a tether cable is studied. Subsequently, an algorithm was developed for waypoint tracking and tests were conducted to verify the performance of the integrated platform. Different from other previously studied cooperation system of USV and AUV, the benefit of the proposed system is real-time motion coordination control between the USV and UUV while transmitting a large amount of data using the tether cable.

In the integrated platform, a catamaran-type USV was designed to overcome the limitations of an ocean environment, whereas the UUV was designed as torpedo-shaped to minimize hydrodynamic resistance. The underwater cable driven by a winch system was installed to supply power from the USV to the UUV and to transmit acquired data from an underwater sonar sensor or camera. Moreover, for fast and accurate coordination of the UUV with respect to the USV, USBL sensor was installed to measure the relative position data of the UUV.

A control algorithm was developed for the integrated platform. The USV has different controllers for distance error and heading angle error with respect to the desired position during waypoint tracking control. Different controller’s output is applied to thrust force by weight scheduling method, which assigns different weights to the powers of the respective controllers according to conditions such as the magnitude of the distance and heading errors with respect to the target position. While USV performing the waypoint tracking control, heading angle tracking, and relative distance control method were employed in the UUV in order to achieve USV–UUV coordination waypoint tracking.

Individual tests were conducted to verify the performance in terms of controlling the USBL and AHRS, which provided the position and heading data of the UUV in the sensors mounted on an actual platform. The results demonstrated the reliability of the sensor measurement. In addition, a number of field tests were performed to verify performance of the control hardware system and designed tracking control algorithm. Firstly, the USV waypoint tracking control test was performed, where results of the test showed that the tracking errors along the path through four designated waypoints were confined to less than 4 m. Secondly, the tracking attitude control test was performed, where the attitude of UUV follows that of the USV quite well with a little time delay. Finally, the relative distance tracking test of the UUV to USV was performed, and the results of relative distance control between the USV and UUV showed that the relative distance was maintained within 5 m under the estimated tidal current of 0.7–0.93 knots, which is acceptable for maritime data application.

## Figures and Tables

**Table 1:** The notation of SNAME for unmanned surface vehicle (USV) and unmanned underwater vehicle (UUV).

**Translational Motion**	**Force**	**Linear Velocity**	**Position**
Surge	*X*	*x*	*u*
Sway	*Y*	*y*	*v*
Heave	*Z*	*z*	*w*
**Rotational Motion**	**Moment**	**Angular Velocity**	**Euler Angle**
Roll	*K*	*ϕ*	*p*
Pitch	*M*	*θ*	*q*
Yaw	*N*	*φ*	*r*

**Table 2:** Coordinates of waypoints for tracking control.

Waypoints	Latitude	Longitude
Waypoint 1	35.076380	129.084503
Waypoint 2	35.076008	129.083708
Waypoint 3	35.074855	129.084510
Waypoint 4	35.075195	129.085328
